# Morphological, structural and physiological differences in heteromorphic leaves of Euphrates poplar during development stages and at crown scales

**DOI:** 10.1111/plb.13078

**Published:** 2020-01-05

**Authors:** J. T. Zhai, Y. L. Li, Z. J. Han, Z. J. Li

**Affiliations:** ^1^ College of Life Science Tarim University/Key Laboratory of Protection and Utilization of Biological Resources in Tarim Basin of Xinjiang Production & Construction Corps Alar People’s Republic of China

**Keywords:** Heteromorphic leaf, morphological anatomy, osmotic regulation, photosynthetic water physiology, *Populus euphratica* Oliv

## Abstract

- Euphrates poplar (*Populus euphratica* Oliv.) has heteromorphic leaves including strip, lanceolate, ovate, and broad‐ovate leaves from base to top in the mature canopy.
- To clarify how diameter at breast height (DBH) and tree height affect the functional characteristics of all kinds of heteromorphic leaves, we measured the morphological anatomical structure and physiological indices of five crown heteromorphic leaves of *P. euphratica *at 2, 4, 6, 8, 10, and 12 m from the same site. We also analysed the relationships between morphological structures and physiological characteristics of heteromorphic leaves and DBH and the height of heteromorphic leaves.
- The results showed that the number of abnormalities regarding blade width, leaf area, leaf thickness, leaf mass per area, cuticle layer thickness, palisade tissue thickness, and palisade tissue/sponge tissue ratio increased with size order and sampling height gradient. Net photosynthetic rate, transpiration rate, stomatal conductance, instantaneous water use efficiency, stable delta carbon isotope ratio, proline and malondialdehyde (MDA) content increased with DBH and sampling height. By contrast, blade length, leaf shape index, and intercellular CO2 concentration decreased with the increase in path order and sampling height gradient. Although MDA content and leaf sponge thickness were not correlated with DBH or sampling height, other morphological structure and physiological parameters were significantly correlated with these variables. In addition, correlations were found among leaf morphology, anatomical structure, and physiological index parameters indicating that they changed with path order and tree height gradient.
- The differences in the morphology, anatomic structure and physiological characteristics of the heteromorphic leaves of*P. euphratica* are related to ontogenesis stage and coronal position.

## Graphical abstract

The differences in the morphological structure and physiological characteristics of the heteromorphic leaves of *P. euphratica* are related to ontogenesis stage and coronal position.

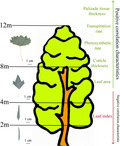

## Introduction

Plants are indicators of environmental change. Leaves are the primary photosynthetic organs of plants and a vegetative organ that is relatively sensitive to environmental changes. Thus, leaf characteristics can reflect the effects of environmental change on plants or plant adaptations to the environment (He *et al.* 2008). In most plants, the same individual will have mature leaves with a completely consistent leaf shape. However, some plants can have mature leaf shapes in the same individual that are not completely consistent, and several different leaf shapes often occur due to differences in growth stage or changes in environmental conditions; this is known as heterophylly. Heterophylly is defined as the development of heteromorphic leaves on the same plant (Bai 2003). This phenomenon involves variations in leaf phenotype, such as variations in size or shape, and occurs along the plant axis (Winn 1999). Since leaf morphology has a considerable effect on leaf functions, such as photosynthesis, transpiration and energy balance (Larcher & Larcher 2003; Leigh *et al.* 2011), heteromorphic leaves that differ in their morphology exhibit differences in functional characteristics that are adapted to environmental stress; thus, heteromorphic leaves can play a crucial role in plant adaptations to environmental change (Winn 1999; Wells & Pigliucci 2000). For example, changes in the leaf morphology of aquatic plants facilitate gas exchange under water (Kuwabara & Nagata 2002; Mommer & Visser 2005). *Sabina vulgaris* grows in a semi‐arid environment, and its scale leaves have higher drought resistance, increased water retention, radiation tolerance and increased water use efficiency (WUE) than needle leaves. However, the accumulation of photosynthetic products is lower in scale leaves than in needle leaves, yet scale leaves are better adapted to arid environments (He & Zhang 2001; Zhang *et al.* 2017). Scale leaves on the outer edge of the crown appear to be more efficient because of their relatively high area‐based photosynthesis rate (*A*), tolerance to photoinhibition and high WUE. Differences in the morphological and physiological characteristics of scale and needle leaves on *S. vulgaris* may be associated with their crown position and growth stage (Tanaka‐Oda *et al.* 2010).

The Euphrates poplar (*Populus euphratica* Oliv.; *Salicaceae*) is found on desert riverbanks in arid and semi‐arid regions around the world. It is an excellent tree species for wind amelioration and sand fixation, as well as for soil and water conservation. The distribution of *P. euphratica* forests on desert riverbanks is related to its dependence on humid environments at the juvenile stage, but the majority of its life cycle takes place in an extremely dry atmosphere and soil environment, where it relies on groundwater obtained from depths of 4–10 m (Huang 1988). *Populus euphratica* grows in heterogeneous habitats and has evolved heterophyllic characteristics associated with ontogenetic development stage (Huang *et al.* 2010; Feng *et al.* 2014). Specifically, strip leaves appear at the seedling stage; lanceolate, ovate and broad‐ovate leaves then appear successively during ontogenetic development. Consequently, mature individuals have strip, lanceolate, ovate and broad‐ovate leaves from the base to the top of the crown. During these different development stages, there are gradual increases in leaf area, number of leaf epithelial and hypodermal cells, epidermal cell length and palisade thickness in *P. euphratica* from the base to the top of the crown (Zhao *et al.* 2016). Studies have shown that the xeric structure of broad‐ovate leaves on the same individual *P. euphratica* tree is more developed than that of lanceolate leaves; furthermore, Rubisco/PEPC is lower and stable carbon isotope ratio (δ^13^C) is higher (Wang *et al.* 1997; Yang *et al.* 2005). Photosynthesis (*A*) and transpiration (*E*) rates of broad‐ovate leaves are always higher than in lanceolate leaves. When leaf temperature is higher, broad‐ovate leaves have enhanced water uptake and reduced temperature by improving *E* for self‐protection (Qiu *et al.* 2005; Zheng *et al.* 2006). In addition, broad‐ovate leaves are relatively resistant to high light intensity and water deficit stress (Bai *et al.* 2011; Wang *et al.* 2014). Both δ^13^C value and WUE of the broad‐ovate leaves are higher than in lanceolate leaves, indicating that the environmental stress intensity of the former is higher than that of the latter (Wang *et al.* 1997; Yang *et al.,* 2005; Ma *et al.,* 2007; Su & Yan 2008). Ability to accumulate osmotic regulatory substances of broad‐ovate leaves is stronger than in lanceolate leaves, indicating that the former are more drought resistant than the latter (Yang *et al.* 2004; Yu *et al.* 2009). The heterophylly of *P. euphratica* is related to development stage (Huang *et al.* 2010; Li *et al.* 2015). Are the differences in morphological structure and physiological characteristics of heteromorphic leaves related to the ontogenetic stage and spatial distribution in the canopy? At present, relevant studies have not been conducted. The purpose of the current study was to comprehensively analyse changes in the morphological structure and physiological characteristics of *P. euphratica* leaves with respect to diameter at breast height (DBH) and tree height to detect changes in these characteristics in relation to individual leaf development stage and crown position.

## Material and Methods

To minimize the environmental effects, we selected *P. euphratica* at different developmental stages but under the same site conditions. The study site is located in a *P. euphratica* forest in the headwater region of the Tarim River on the north‐western margin of the Tarim Basin (81°17'56.52″ E, 40°32'36. 90″ N, 980 m a.s.l.). This region has a hot, dry summer with dry weather and little rainfall throughout the year. The average annual precipitation is 50 mm, potential evaporation is 1900 mm and temperature of 10.8 °C, with average annual sunshine hours of 2900 h, making it a typical temperate desert climate. The *P. euphratica* forest is 500 m from the Tarim River, and groundwater level there (1.0–1.5 m) is suitable for growing *P. euphratica*.

### Experimental design and sampling

The *P. euphratica* forest at the study site covers an area of 180.6 ha, with 355 individual *P. euphratica* trees. Using DBH and 4 cm as the interval, the *P. euphratica* forest can be divided into five diameter classes, 4, 8, 12, 16 and 20 cm. Three sample trees with uniform crowns were selected from each diameter class, with 15 trees in total (Table S1). Sampling points were selected from the trunk base of the sample trees along the tree height (H) at 2‐m intervals. These points were distributed at 2, 4, 6, 8, 10 and 12 m of the sample trees, and also the height of heteromorphic leaves in the vertical space of the canopy. At each sampling point, 1‐year‐old branches were collected from each of the four directions (east, south, west and north). Thirty branches were collected at each point, and sample leaves were taken at the fourth node from the base of the branch. A total of 30 leaves per sampling point were obtained to analyse leaf morphology, anatomy, dry mass, δ^13^C and concentrations of proline (Pro) and malondialdehyde (MDA). The leaves used for the Pro and MDA analysis were stored in liquid nitrogen after collection.

### Measurements of leaf morphological and anatomical parameters

We used the blade length‐to‐width ratio (leaf index) to assess changes in leaf shape. The blade length, blade width (BW) and leaf area were measured using a scanner (MRS‐9600TFU2) and la‐s plant image analysis software. The leaf index was calculated from the blade length/blade width ratio (Jiang & He 2014).

The blade was cut transversely at its widest part. The material that retained the primary vein and leaf margin was selected and fixed in a formalin–acetic acid–alcohol (FAA) solution. Tissue sections were prepared as 8‐μm thick paraffin sections, double‐stained with sarranine–fast green and mounted in a neutral resin. The leaf thickness, cuticle thickness, palisade tissue thickness and spongy tissue thickness were measured under a Leica microscope (Leica DM4 B, Wetzlar, Germany). The palisade tissue/sponge tissue ratio (PSR) was calculated. Five fields of view were observed for each leaf, and 20 values were obtained for each field of view. The average values for the leaf structural parameters in five fields of view were collected as the anatomical parameters of each leaf.

### Measuring the leaf dry mass

The leaves collected at different sampling points were kept in paper bags and placed in a pre‐heated oven. The oven temperature was raised to 105 °C for a 10‐min deactivation of the samples. The oven temperature was then lowered to 65 °C, at which the samples were dried to constant weight. The paper bags containing the material were removed and placed in a desiccator. After being cooled to room temperature, the samples were weighed on an electronic balance (0.001 g). The leaf mass per area (LMA) was calculated based on the leaf area and dry mass.

### Measurement of leaf photosynthesis

The leaf photosynthetic parameters were measured from 11:00–13:00 h on sunny and cloudless days in mid‐July. One‐year‐old branches were collected with pruning shears and immediately wrapped in plastic wrap to cover the incision. Leaves were collected at the fourth node from the base of the branch, and a Li‐6400 IRGA was used to measure gas exchange parameters, namely, net *A*, *E*, stomatal conductance (*g_s_*) and intercellular CO_2_ concentration (*C_i_*). The instantaneous water use efficiency (WUE*_i_*) of the leaves was calculated as WUE*_i_* = *A*/*E*. Ten leaves from each sampling point were measured, with three replicates.

### Measuring the stable δ^13^C

After measuring leaf morphological parameters for each sample tree, we immediately rinsed the samples in distilled water and then deactivated them in an oven at 105 °C. After being air‐dried, the samples were oven‐dried at 60 °C for 48 h to a constant weight. The dry samples were pulverized with a pulverizer and passed through a 90‐mesh sieve. For carbon isotope analysis, plant samples were prepared using a glass vacuum system. The combustion furnace was connected to the power supply and the furnace temperature was maintained at 1000 °C. After being vacuumed, the system was supplied with O_2_. A porcelain spoon containing a *P. euphratica* leaf sample was placed inside the combustion tube and combusted in the high‐temperature zone for 2 min. The CO_2_ gas was then collected and purified by freezing, and the purified gas analysed for its carbon isotope composition using a stable gas isotope mass spectrometer.

### Measurements of MDA and Pro concentrations

The leaf Pro concentration was measured using the acid ninhydrin method. In brief, 0.5–1.0 g of each fresh sample was weighed into a mortar or homogenizer, and then 3 ml 80% ethanol and a small amount of quartz sand were added. After the sample was ground, the resulting homogenate was transferred into a graduated test tube with a plug. The mortar was rinsed with 80% ethanol and the rinsing liquid combined with the homogenate to a final volume of 10 ml. Each tube was plugged and kept in a boiling water bath for 10 min. After that, 0.25 g activated carbon powder was added to the tube and the mixture shaken and filtered. An aliquot of 10 ml sample extract and 1 g artificial zeolite were added to the tube, which was shaken for 10 min. The zeolite was then removed by filtration. Furthermore, 2 ml of the abovementioned sample filtrate, 2 ml glacial acetic acid and 2 ml ninhydrin reagent were added to the tube, which was shaken, plugged and kept in a boiling water bath for 10–15 min. After cooling, the solution was subjected to a colorimetric assay at 515 nm and the Pro concentration calculated.

The leaf MDA concentration was measured using thiobarbituric acid colorimetry. In brief, 1 g leaf sample was weighed and cut into small pieces. This sample was ground with 2 ml 10% trichloroacetic acid (TCA) and a small amount of quartz sand. Then, 8 ml 10% TCA were added, and the sample fully ground. The homogenate was centrifuged at 1776 *g* for 10 min, and the supernatant collected as the sample extract. An aliquot of 2 ml extract was taken and mixed with 2 ml 0.6% thiobarbituric acid. The mixture was allowed to react in a boiling water bath for 15 min and then rapidly cooled and centrifuged. The optical density of the supernatant was measured at 532, 450 and 600 nm, and the results used to calculate the MDA concentration.

### Statistical analysis

A one‐way anova was performed using dps 7.05 to analyse relationships among tree height and leaf shape, morphology, structure, gas exchange capacity and WUE. The significance of differences was determined at α = 0.05. A correlation analysis was also performed to test for correlations between the parameters. All data were normal distributions and produced single peaks.

## Results

### Changes in leaf morphology with DBH and height of heteromorphic leaves

**Figure 1:**
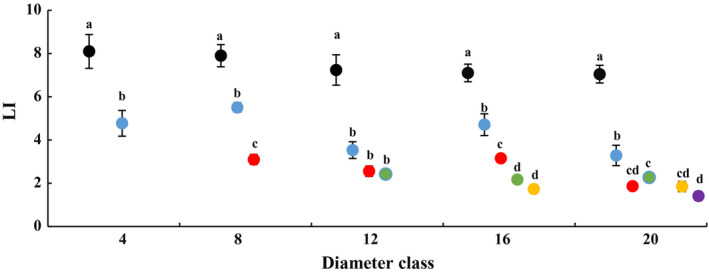
Changes in leaf index (LI) across diameter class and sampling height scales on *P. euphratica.* Black dots indicate sampling height at 2 m, blue dots indicate 4 m, red dots indicate 6 m, green dots indicate 8 m, yellow dots indicate 10 m, purple dots indicate 12 m.

**Figure 2:**
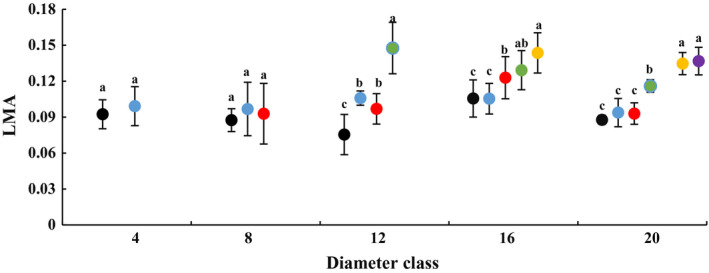
Changes in leaf mass per area (LMA) across diameter class and sampling height scales on *P. euphratica.* Black dots indicate sampling height at 2 m, blue dots indicate 4 m, red dots indicate 6 m, green dots indicate 8 m, yellow dots indicate 10 m, purple dots indicate 12 m.

In each diameter class, blade length and leaf index gradually decreased with increasing tree height, while blade width, leaf area, leaf thickness and LMA increased. There were significant differences in leaf morphological characteristics across various heights (Figs 1, 2; Figures S1–S4). Compared with those at a height of 2 m, the heteromorphic leaves clearly became larger, thicker and denser at the maximum sampling height of each diameter class, with pronounced changes in leaf morphology. For example, in class 4, an increase in height from 2 to 4 m resulted in a 19.2% decrease in blade length, a 71.4% increase in blade width, a 14.6% increase in leaf area and a 23.45% increase in leaf thickness. Based on the result that leaf index decreased by 41.1%, the leaf shape at a height of 4m was deemed to be a strip leaf. In class 20, an increase in height from 2 to 12 m caused a 33.3% decrease in blade length, a 386% increase in blade width, a 157% increase in leaf area, a 72% increase in leaf thickness and a 62.5% increase in LMA. That means the morphological index was significantly different at 2 m in class 20, and also at 12 m in that class. Based on the leaf index result, leaf shape was deemed to be a strip leaf at the 2‐m height, a lanceolate leaf at 4‐m height, an ovate leaf at 6–10 m height, and a broad‐ovate leaf at 12‐m height. These results indicate that the morphology of heteromorphic leaves changed with increasing DBH and height of heteromorphic leaves. These changes differed across diameter class and crown height gradients in each diameter class.

Changes were also observed across diameter class of each crown scale (Table S3). The blade length increased, while leaf index, leaf area and leaf thickness decreased with increasing diameter class from class 4 to 20 at 2 m. These indices differed significantly between classes 4 and 20. The blade width increased, while leaf index and leaf thickness decreased from class 4 to 20 at 4 m, and these indices differed significantly between the first and last class. The blade width and leaf area increased, and blade length and leaf index decreased from class 8 to 20 at 6 m, and these indices differed significantly between class 8 and 20. The blade length, leaf thickness and LMA decreased from class 12 to 20 at 8 m, and these indices differed significantly between class 12 and 20. These results show that the morphology of heterophylly differed in distinct diameter classes at the same crown scale.

### Changes in leaf anatomical structure with DBH and height of heteromorphic leaves

**Figure 3:**
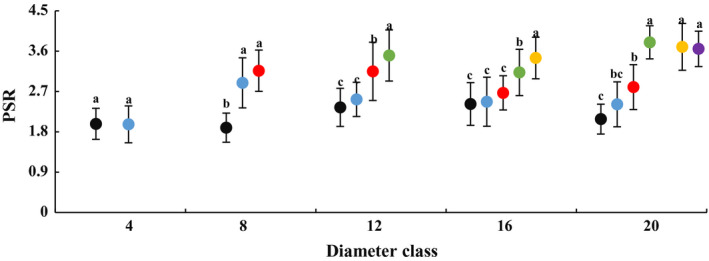
Changes in leaf palisade tissue/spongy tissue ratio (PSR) across diameter class and sampling height scales in *P. euphratica.* Black dots indicate sampling height at 2 m, blue dots indicate 4 m, red dots indicate 6 m, green dots indicate 8 m, yellow dots indicate 10 m, purple dots indicate 12 m.

**Figure 4:**
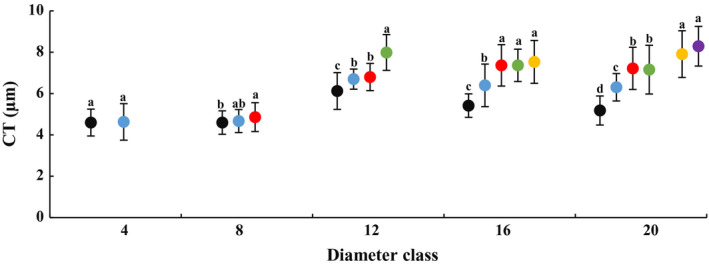
Changes in leaf cuticle thickness (CT) across diameter class and sampling height scales in *P. euphratica.* Black dots indicate sampling height at 2 m, blue dots indicate 4 m, red dots indicate 6 m, green dots indicate 8 m, yellow dots indicate 10 m, purple dots indicate 12 m.

In each diameter class, palisade tissue thickness, PSR and cuticle thickness increased with increasing tree height, and leaf anatomical structure significantly differed at various heights (Figs 3, 4; Figures S5, S6). Compared with results at a height of 2 m, the heteromorphic leaf palisade tissue and cuticle appeared to be thicker, while PSR was larger at the maximum sampling height of each diameter class. For example, in class 4, with an increase in height from 2 to 4 m, leaf palisade tissue thickness, spongy tissue thickness, cuticle thickness and PSR all increased by 42.93%, 11.34%, 0.65% and 0.5%, respectively. In class 20, as height increased from 2 to 12 m, leaf palisade tissue thickness, spongy tissue thickness, cuticle thickness and PSR also increased by 78.31%, 6.10%, 60.15% and 75.48%, respectively. In this class, the cuticle thickness, palisade tissue thickness and PSR of heteromorphic leaves at 12 m was significantly different to the results at 2 m, as was spongy tissue thickness of heteromorphic leaves at 6 m. These results indicate that leaf anatomical structure became more xerophytic with increasing DBH and height gradient of heteromorphic leaves. These changes varied with diameter class and crown height gradient in each diameter class.

The comparisons also showed that the anatomy of heteromorphic leaves differed across diameter classes at the same crown scale (Table S4). The spongy tissue thickness, PSR and cuticle thickness first increased and then decreased from class 4 to 20 at 2 m. Over the classes, the difference in palisade tissue thickness was not significant, and spongy tissue thickness at class 8, PSR and cuticle thickness at class 12 were significantly higher than at classes 4 and 20. The palisade tissue thickness and spongy tissue thickness decreased, and PSR and cuticle thickness increased from class 4 to 20 at 4 m. These indices were significant different between the first and the last class. The palisade tissue thickness of class 16 was significantly lower than that of class 12, and the difference in spongy tissue thickness was not significant from class 4 to 20 at 6 or 8 m. The difference in spongy tissue thickness from class 20 was significantly lower than that from class 16 at 12 m but was not significant for palisade tissue thickness.

### Changes in leaf photosynthetic capacity with DBH and height of heteromorphic leaves

**Figure 5:**
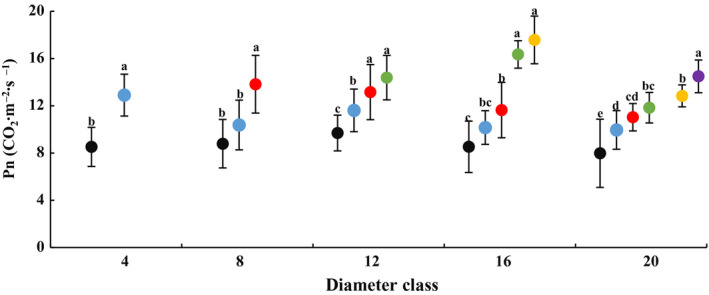
Changes in leaf net photosynthesis rate (*A*) across diameter class and sampling height scales in *P. euphratica.* Black dots indicate sampling height at 2 m, blue dots indicate 4 m, red dots indicate 6 m, green dots indicate 8 m, yellow dots indicate 10 m, purple dots indicate 12 m.

In each diameter class, leaf *A* gradually increased with increasing tree height. The *A* of the leaves at the maximum sampling height of each diameter class was significantly different compared with that of the 2‐m height. No significant differences were observed in leaf *E* for classes 4, 8, 12 and 16; nonetheless, in class 20, *E* gradually increased with increasing height, and an extremely significant difference was observed in *E* between the 12‐m and 2‐m heights (Fig. 5; Figure S7). Leaf *C_i_* decreased with increasing sampling height in classes 12, 16 and 20, and *C_i_* at the maximum sampling height of each class was significantly lower than that at 2‐m height. The largest change was found in class 20, with a 42.55% decrease in *C_i_* (Figure S8). The heteromorphic leaf *g_s_* generally increased with increasing tree height in classes 8, 12, 16 and 20 (Figure S9), while the index at the maximum sampling height was significant higher than at 2 m in classes 8, 12 and 16. But the most pronounced change occurred in class 16, with an increase from 0.11 at 2‐m height to 0.24 at 10‐m height, an increase of 118.18%. These results indicate that the photosynthetic capacity of the heteromorphic leaves increased with increasing DBH and height gradient of the heteromorphic leaves. These changes differed across diameter class and crown height gradients at each diameter class.

The above comparisons also indicated that the differences in leaf photosynthetic capacity differed across diameter classes at the same crown scale (Table S5). The *A* and *E* first increased and then decreased from class 4 to 20 at 2 m. Within these classes, *A* of class 12 and *E* of class 8 were sharply higher than for class 4 and 20. The *A* decreased, *E* and *C_i_* increased from class 4 to 20 at 4 m. At crown height, the difference in *g_s_* across diameter classes was not significan; *A* of class 20 was significantly lower than that in class 4, but *E* and *C_i_* of this class were sharply higher than in class 4. The *A*, *E*, *C_i_* and *g_s_* of class 16 were sharply higher than in class 12 at 8 m. Compared with the results of class 16, *A* and *g_s_* of class 20 at 10 m were significantly lower and *E* was sharply higher.

### Changes in leaf WUE_i_ with DBH and height of heteromorphic leaves

**Figure 6:**
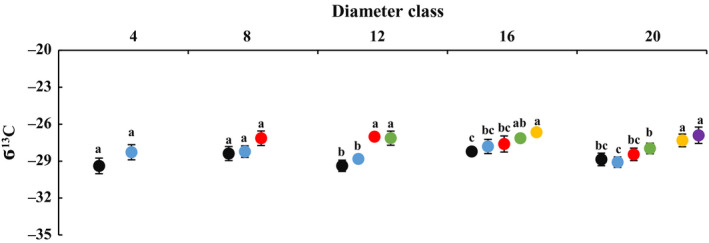
Changes in leaf carbon isotope value (δ^13^C) across diameter class and sampling height scales in *P. euphratica.* Black dots indicate sampling height at 2 m, blue dots indicate 4 m, red dots indicate 6 m, green dots indicate 8 m, yellow dots indicate 10 m, purple dots indicate 12 m.

**Figure 7:**
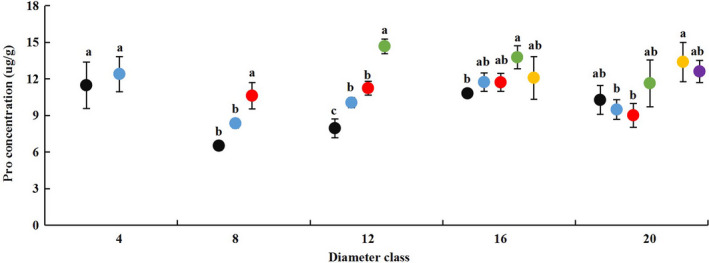
Changes in leaf proline (Pro) concentration across diameter class and sampling height scales in *P. euphratica.* Black dots indicate sampling height at 2 m, blue dots indicate 4 m, red dots indicate 6 m, green dots indicate 8 m, yellow dots indicate 10 m, purple dots indicate 12 m.

The WUE*_i_* of leaves increased with the increasing sampling height gradient in each diameter order, and the difference between the highest sampling point of each diameter order and a height of 2 m was significant (Fig. 6). Among these results, the increase in leaf WUE*_i_* in class 16 was largest at 93.39% (Figure S10). No significant difference was found in δ^13^C value with regard to height gradients of diameters 4 and 8; however, δ^13^C increased with sample height gradients in diameters 12, 16 and 20, and maximum δ^13^C was significantly higher than at 2 m (Fig. 7). These results show that WUE*_i_* of the heteromorphic leaves increased with DBH and height of heteromorphic leaves. These changes also varied with diameter class and crown height gradients at each diameter order.

The comparisons showed that leaf WUE*_i_* was different across diameter classes at the same crown scale (Table S6). The value of δ^13^C increased at 2 m and decreased at 4 m from class 4 to 20. Within the classes of the two scales, the difference in δ^13^C was significant between class 4 and 20. The leaf WUE*_i_* and δ^13^C of class 16 were sharply higher than in class 20.

### Changes in leaf Pro and MDA concentrations with DBH and height of heteromorphic leaves

The leaf Pro concentration generally exhibited an increasing trend with sampling height in different diameter classes, except for class 4 (Fig. 7). The Pro concentration at each maximum height of the five diameter classes increased by 8.1%, 62.98%, 84.52%, 11.85% and 22.78% compared with that at 2‐m height. Compared with leaf Pro concentration at 2 m, there was a significant difference that 6 m in class 8, 8 m in class 16 and 10 m in class 20. Similarly, leaf MDA concentration also showed an increasing trend with sampling tree gradient in various diameter classes, except for class 12 (Figure S11). Leaf MDA concentration at maximum height in the five diameter classes increased by 21.6%, 13.88%, 10.71, 26.92% and 17.64% compared with 2‐m height. The comparison also showed significant differences for 4 and 6 m in class 8, 8 and 10 m in class 16, and 4 m, 8, 10 and 12 m in class 20.

The comparisons of leaf Pro and MDA concentrations indicated that stress resistance of heteromorphic leaves differed across diameter classes at the same crown scale (Table S7). Concentrations of Pro and MDA first decreased and then increased from class 4 to 20 at 2 m. At the same scale, concentrations at class 12 were sharply lower than at class 4 and 20. The Pro concentration of class 20 at 8 m was significantly lower and MDA was sharply higher than for class 16. The MDA concentration of class 20 at 10 m was sharply higher than at class 16, while there was no significant difference in Pro.

### Correlations among leaf morphology, structure and physiological characteristics and DBH and height of heteromorphic leaves

As Table 1 shows, blade width, leaf area, leaf thickness, LMA, palisade tissue thickness, cuticle thickness and PSR were positively correlated with DBH and height of heteromorphic leaves, whereas blade length and leaf index were negatively correlated with DBH and height of heteromorphic leaves. The results show that the morphological and anatomical structural changes in heteromorphic leaves of *P. euphratica* were closely related to changes in DBH and height of heteromorphic leaves.

**Table 1:** Correlation analysis between morphological and anatomical structure parameters in heteromorphic leaves with the DBH and tree height of *P. euphratica*

	BL	BW	LI	LA	LT	LMA	PT	ST	CT	PSR
Sampling height	−0.57**	0.93**	−0.83**	0.85**	0.82**	0.84**	0.85**	−0.25	0.82**	0.88**
DBH	−0.93**	0.97**	−0.79*	0.93**	0.93**	0.94**	0.92**	−0.66	0.96**	0.91**

N = 220; BL = blade length; BW = blade width; LI = leaf index; LA = leaf area; LT = leaf thickness; LMA = LEAF mass per area; PT = palisade tissue thickness; ST = spongy tissue thickness; CT = cuticle thickness; PSR = palisade tissue/spongy tissue ratio.

* *P* < 0.05,

** *P* < 0.01.

Table 2 shows that *A*, *E*, δ^13^C and Pro content were positively related to height of heteromorphic leaves and DBH, and negatively correlated with *C_i_*. The height of heteromorphic leaves, but not DBH, were positively related to *g_s_* and WUE*_i_*. These results show that the photosynthetic capacity, WUE and Pro metabolism of the heteromorphic leaves were closely related to changes in DBH and the height of heteromorphic leaves.

**Table 2:** Correlation analysis between physiological parameters of heteromorphic leaves and DBH and tree height of *P. euphratica*

	*A*	*E*	*g_s_*	*C_i_*	WUE*_i_*	δ^13^C	Pro	MDA
Sampling height	0.83**	0.51*	0.48*	−0.58**	0.49*	0.79**	0.64**	0.31
DBH	0.86*	0.89**	0.72	−0.96**	0.33	0.94**	0.85*	0.67

N = 180; *A *= Photosynthetic rate; *E* = Transpiration rate; *g_s_* = Stomatal conductance; *C_i_* = Intercellular CO_2_ concentration; WUE*_i_* = Instantaneous water use efficiency; Pro = Proline; MDA = Malondialdehyde.

* *P* < 0.05,

** *P* < 0.01.

Leaf area, leaf thickness and leaf anatomy form the basis for drought resistance and photosynthesis. The analysis in Table S2 revealed correlations among the morphological, structural and physiological parameters of heteromorphic leaves of *P. euphratica*, which showed coordinated changes. The preliminary results showed that the increases in leaf area, leaf thickness, palisade tissue thickness and PSR increased the photosynthetic area, and that increase in PSR benefitted photosynthesis, increasing *A* of the profiled leaves. Increases in leaf area improved WUE and promoted *A* and *E*. Increases in *E* increased the water absorption of the leaves at the top of the tree. With increases in leaf area, Pro content increased water retention and stress tolerance of the large leaves in the upper portion of the canopy.

## Discussion

### Morphological and structural adaptive characteristics of the heteromorphic leaves during development stages and at crown scales

The special adaptive structure of plant leaves includes leaf morphology, thickness, surface features and other anatomical features (Fang *et al.* 2000). The thicker the leaves, the higher the water storage capacity and drought tolerance of the plants (Chen *et al.* 2001). Cuticle thickness is directly related to plant water retention capacity and can reduce water loss (Li *et al.* 1996). Highly developed palisade tissue not only prevents the ‘burning’ of the mesophyll by intense light in arid regions, but also effectively uses diffracted light for photosynthesis (Yan et al. 2000). Differences in the specific adaptive structure of leaves may be attributed to the location of the same species under different ecological conditions or may be linked to leaf position during the crown and growth stages (England & Attiwill 2006; He *et al.* 2008; Tanaka‐Oda *et al.* 2010). For example, England & Attiwill (2006) found that leaf area and stomatal size decreased with increasing tree height, whereas epidermal thickness and stomatal density increased. He *et al. *(2008) also found that leaf area decreased with the increasing tree height of *Parashorea chinensis*, whereas stomatal density and leaf mass per area increased. Additionally, the above study indicated that leaf anatomical structure, including the palisade tissue thickness, epidermal thickness and cuticle thickness, PSR and stomatal and vascular bundle density, will all increase with increasing tree height, and that tree height is significantly correlated with these leaf parameters. It was therefore considered that morphology and anatomical structure of *P. chinensis* leaves would become more xeromorphic as tree height increased (He *et al.* 2008). This conclusion supports to the hypothesis that the effects of tree height (gravity and hydraulic resistance) may gradually increase water stress depending on tree height (Ryan & Yoder 1997). Our results are similar to the above results. *Populus euphratica* abnormal foliage thickness, leaf area, specific leaf weight, cuticle thickness, main tissue thickness and PSR increased with increasing DBH and tree height of heteromorphic leaves. These indices were significantly related to DBH and tree height of *P. euphratica*, which indicates development of a stronger xerophytic structure*.* These morphological, structural and functional differences were related to individual development stages and canopy position in which the heteromorphic leaves were located. Previous studies have shown that the difference in xylem pressure across the height of the tree affects morphological structure of the leaves at the corresponding tree height (*i.e.* water potential in the xylem of the tree body decreases as tree height increases). The upper leaves of the tree body can only reduce water loss through more obvious drought‐resistant structures to cope with water stress caused by the height of the tree (Ryan & Yoder 1997; He *et al.* 2008). We conclude that the difference in morphological and structural characteristics of the heteromorphic leaves of *P. euphratica* during the ontogenetic stages and crown scales is an adaptation to environmental drought stress and water stress caused by tree height.

### Adaptive characteristics shown by the difference of photosynthetic capacity in heteromorphic leaves during developmental stages and at crown scales

Currently, the hypothesis on height limitations is primarily focused on the increase in limitations to water transport in tall trees and the resulting reduction in leaf photosynthesis (Ryan & Yoder 1997). When trees grow taller, even if soil humidity is adequate, the increased leaf water stress due to gravity and path length resistance may eventually limit leaf expansion and photosynthesis needed for further growth (Koch *et al.* 2004). Previous studies have shown that on the same individual tree of *P. euphratica*, broad‐ovate leaves with a high *A* and WUE distributed in the upper part of the crown are more resistant to atmospheric drought than lanceolate leaves distributed in lower parts of the crown (Qiu *et al.* 2005; Zheng *et al.* 2006; Bai *et al.* 2011; Wang *et al.* 2014). Preliminary data indicate that these differences in photosynthetic capacity of heteromorphic leaves may be associated with the crown position of the heteromorphic leaves. Our research found that *P. euphratica* leaf shape photosynthetic capacity, from class 4 to 20, is characterized by obvious differences with increasing sampling height. Leaf *A*, *E* and *g_s_* increase with tree height gradient, and *C_i_* decreases with increasing sampling height. These physiological parameters are significantly related to sampling height in the crown, DBH and the morphological and structural indices. These results show that differences in physiological characteristics are related to not only changes in ontogenetic stage and tree height but also to changes in the morphological structure of heteromorphic leaves. Studies have reported that the branches of *P. euphratica* at the base of the canopy are the longest and thinnest but have the most leaves per branch and the smallest leaf area, whereas branches at the top of the canopy are the shortest and thickest but have the fewest leaves per branch and the largest leaf area (Li *et al.* 2015). Branches tend to become shorter and coarser, and the ontogenetic stages and tree height gradients change with fewer leaves per branch to ensure growth of fewer large leaves, whereas the large, thick leaves with well‐developed branching are the basis for enhancing transpiration tension and increasing the photosynthetic area.

Unlike the leaf area decrease with increasing tree height observed for *P. chinensis* (He *et al.* 2008), heteromorphic leaves of *P. euphratica* showed an increased leaf area and enhanced photosynthetic capacity with increasing sampling height. One possible reason is that the leaf area increase resulting from the sampling height increase improved *E* and transpirational pull, which are conducive to the rise of water. As such, expansion of the leaf area would not be limited, while a large leaf area could ensure an increase in *A* and *E*. Moreover, leaf mass to area is a parameter that measures the performance of leaf photosynthesis. Leaf mass to area is related to leaf photosynthesis, leaf area index, leaf nitrogen concentration and leaf development. Leaf mass to area increased with increasing sampling height. A high leaf mass to area can accumulate more photosynthetic products, increase tissue density and biomass, and drive heteromorphic leaves to produce sufficient organic matter for their own growth.

### Ecological implications indicated by WUE*_i_* of heteromorphic leaves with increasing DBH and height of heteromorphic leaves

Leaf WUE*_i_* and δ^13^C are important indicators of water use efficiency, especially δ^13^C, which is used to measure long‐term water use efficiency of plants. Numerous studies have shown that drought significantly increases WUE*_i_*, whereas high temperatures significantly reduce it (Yan *et al.* 1998; Gratani *et al.,* 2000; Kellomaki & Wang, 2001; Vu *et al.,* 2002; Zhang *et al.* 2004; Yin *et al.,* 2005; Monclus *et al.,* 2006; Xu *et al.* 2008). These papers studied spatial changes and interspecific variations of leaf δ^13^C in temperate deciduous forest and concluded that the upper leaves of trees had higher δ^13^C values than the lower leaves. In our study, we found that WUE*_i_* and δ^13^C of heteroform leaves of *P. euphratica* both increased with the sampling height gradient, and were positively and significantly correlated with sampling height, indicating that the height of heteromorphic leaves affects WUE of heteromorphic leaves, which is consistent with previous findings (Sternberg *et al.,* 1989; Yan *et al.,* 1998; Koch *et al.,* 2004; He *et al.* 2008). The results also showed that the δ^13^C increased with DBH, beginning with the large (class 12) diameter order; δ^13^C was also significantly and positively correlated with leaf area, leaf thickness and leaf mass to area. Obviously, the increase in δ^13^C with DBH and height of heteromorphic leaves is based on changes in morphological structure of the heteromorphic leaves associated with these changes. Higher leaf mass to area leads to accumulation of photosynthetic products as well as increases in tissue density and biological mass; together, these effects increase intrinsic resistance to carbon dioxide diffusion, reduce photosynthesis and increase the water limit, thereby increasing δ^13^C (Koch *et al.* 2004). The heterophyll δ^13^C values associated with individual development and crown position are related to the heterophyll xerophytic structural differences in physical performance. Increases in DBH and tree height of *P. euphratica* affect large (broadly ovate) leaves, thereby increasing individual WUE. These findings have extremely important biological significance for woody plants in dry desert environments.

### Adaptive characteristics indicated by the osmotic substance concentration of heteromorphic leaves with increasing DBH and height of heteromorphic leaves

Plants have evolved complex mechanisms to sense and adapt to water deficits. For example, plants can cope with stress by maximizing water absorption and minimizing water loss or accumulating osmotic substances, thus preventing drought (Ma *et al.* 2014). Malondialdehyde content is commonly used to assess redox and osmoregulation, and plants with high drought resistance show small increases in MDA concentration (Gomezdelcampo *et al.* 2002). Plants with low MDA concentrations have high antioxidant capacity, reflecting their high drought resistance (Apel & Hirt 2004). Here, we found that there was little increase in MDA concentration of heteromorphic leaves with increasing tree height in *P. euphratica* across various diameter classes. This finding implies that the heteromorphic *P. euphratica* leaves maintained a relatively high antioxidant capacity with increases in morphological parameters and leaf area when tree height increases. This capacity was not correlated with tree height. Proline is a plant osmotic substance that is relatively sensitive to stress. The proline concentration is directly proportional to drought resistance in plants (Singh *et al.* 1972; Verbruggen & Hermans 2008). Our current results showed that proline content increased with the diameter order and sampling height gradient. Furthermore, proline content was significantly and positively correlated with diameter class, sampling height and leaf area. These findings indicate that proline regulates drought resistance of *P. euphratica*, which increases with ontogenetic stage and crown scale. Proline content also increased with leaf area, which enhanced water retention and stress resistance of the large leaves located in the upper portion of the canopy. According to the current analysis, the content of proline in heteromorphic leaves of *P. euphratica* was synergistic with DBH, tree height and area of the heteromorphic leaves to adjust drought resistance of the different development stages and coronal parts of *P. euphratica*. Thus, an adaptive strategy of *P. euphratica* is used to cope with water stress caused by environmental drought and tree height.

## Conclusions

Our results revealed that *P. euphratica* heterophyll morphological structure and physical characteristics showed obvious differences through crown scales of each ontogenetic stage. With increasing diameter class and crown scale, the morphology, structure and physiological characteristics of heteromorphic leaves became more obviously xerophytic, with higher photosynthetic capacity, WUE, drought resistance and water retention ability to adapt to water stress derived from increasing environmental drought and tree height.

## Supporting information


**Figure S1.** Changes in leaf blade length across diameter class and sampling height scales in *P. euphratica.* Black dots indicate sampling height at 2 m, blue dots indicate 4 m, red dots indicate 6 m, green dots indicate 8 m, yellow dots indicate 10 m, purple dots indicate 12 m.

**Figure S2.** Changes in leaf blade width across diameter class and sampling height scales in *P. euphratica.* Black dots indicate sampling height at 2 m, blue dots indicate 4 m, red dots indicate 6 m, green dots indicate 8 m, yellow dots indicate 10 m, purple dots indicate 12 m.

**Figure S3.** Changes in leaf area across diameter class and sampling height scales in *P. euphratica.* Black dots indicate sampling height at 2 m, blue dots indicate 4 m, red dots indicate 6 m, green dots indicate 8 m, yellow dots indicate 10 m, purple dots indicate 12 m.

**Figure S4.** Changes in leaf thickness across diameter class and sampling height scales in *P. euphratica.* Black dots indicate sampling height at 2 m, blue dots indicate 4 m, red dots indicate 6 m, green dots indicate 8 m, yellow dots indicate 10 m, purple dots indicate 12 m.

**Figure S5.** Changes in leaf palisade thickness across diameter class and sampling height scales in *P. euphratica.* Black dots indicate sampling height at 2 m, blue dots indicate 4 m, red dots indicate 6 m, green dots indicate 8 m, yellow dots indicate 10 m, purple dots indicate 12 m.

**Figure S6.** Changes in leaf spongy tissue thickness across diameter class and sampling height scales in *P. euphratica.* Black dots indicate sampling height at 2 m, blue dots indicate 4 m, red dots indicate 6 m, green dots indicate 8 m, yellow dots indicate 10 m, purple dots indicate 12 m.

**Figure S7.** Changes in leaf transpiration rate (*E*) across diameter class and sampling height scales in *P. euphratica.* Black dots indicate sampling height at 2 m, blue dots indicate 4 m, red dots indicate 6 m, green dots indicate 8 m, yellow dots indicate 10 m, purple dots indicate 12 m.

**Figure S8.** Changes in leaf intercellular CO_2_ concentration (*C_i_*) across diameter class and sampling height scales in *P. euphratica.* Black dots indicate sampling height at 2 m, blue dots indicate 4 m, red dots indicate 6 m, green dots indicate 8 m, yellow dots indicate 10 m, purple dots indicate 12 m.

**Figure S9.** Changes in leaf stomatal conductance (*g_s_*) across diameter class and sampling height scales in *P. euphratica.* Black dots indicate sampling height at 2 m, blue dots indicate 4 m, red dots indicate 6 m, green dots indicate 8 m, yellow dots indicate 10 m, purple dots indicate 12 m.

**Figure S10.** Changes in leaf instantaneous water use efficiency (WUE*_i_*) across diameter class and sampling height scales in *P. euphratica.* Black dots indicate sampling height at 2 m, blue dots indicate 4 m, red dots indicate 6 m, green dots indicate 8 m, yellow dots indicate 10 m, purple dots indicate 12 m.

**Figure S11.** Changes in leaf malondialdehyde (MDA) concentration across diameter class and sampling height scales in *P. euphratica.* Black dots indicate sampling height at 2 m, blue dots indicate 4 m, red dots indicate 6 m, green dots indicate 8 m, yellow dots indicate 10 m, purple dots indicate 12 m.

**Table S1.** Information about the sampled trees.

**Table S2.** Correlation analysis among parameters related to morphological structure and physiology of heteromorphic leaves in *P. euphratica*.

**Table S3.** Comparisons of morphology in heteromorphic leaves in sampling height gradients in the same diameter class and across diameter class at the same height.

**Table S4.** Comparisons of anatomical structure of heteromorphic leaves in sampling height gradients in the same diameter class and across diameter class at the same height.

**Table S5.** Comparisons of gas exchange capacity of heteromorphic leaves in sampling height gradients in the same diameter class and across diameter class at the same height.

**Table S6.** Comparisons of water use efficiency of heteromorphic leaves in sampling height gradients in the same diameter class and across diameter class at the same height.

**Table S7.** Comparisons of physiological characteristics of heteromorphic leaves in sampling height gradients in the same diameter class and across diameter class at the same height.
