# Supplementary figures and images for: Morphological, structural and physiological differences in heteromorphic leaves of Euphrates poplar during development stages and at crown scales
Source: Plant Biol (Stuttg). 2020 Jan 5;22(3):366–75. doi: 10.1111/plb.13078 (PMC7318281; doi:10.1111/plb.13078)

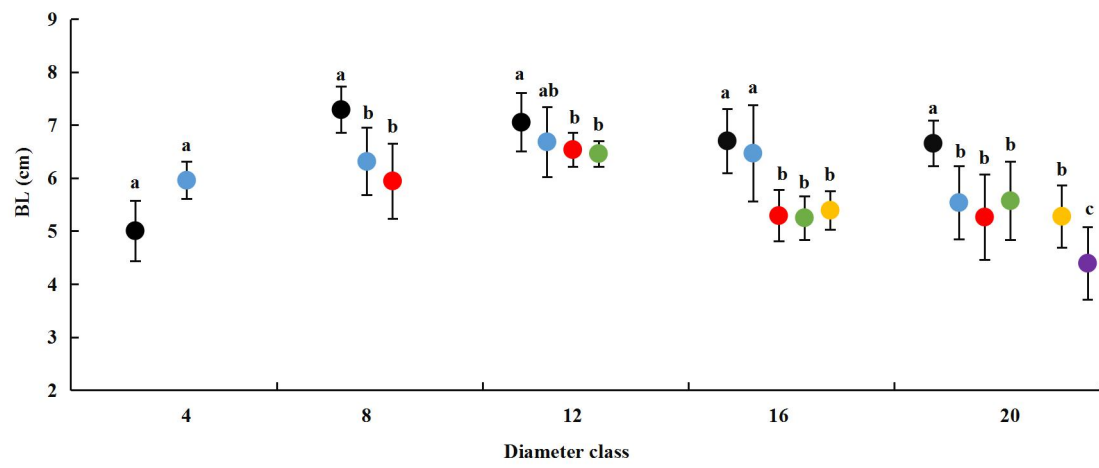

Supplement: Supplementary file 1 — Figure S1. Changes in leaf blade length across diameter class and sampling height scales in P. euphratica. Black dots indicate sampling height at 2 m, blue dots indicate 4 m, red dots indicate 6 m, green dots indicate 8 m, yellow dots indicate 10 m, purple dots indicate 12 m. [file PLB-22-366-s001.pdf]

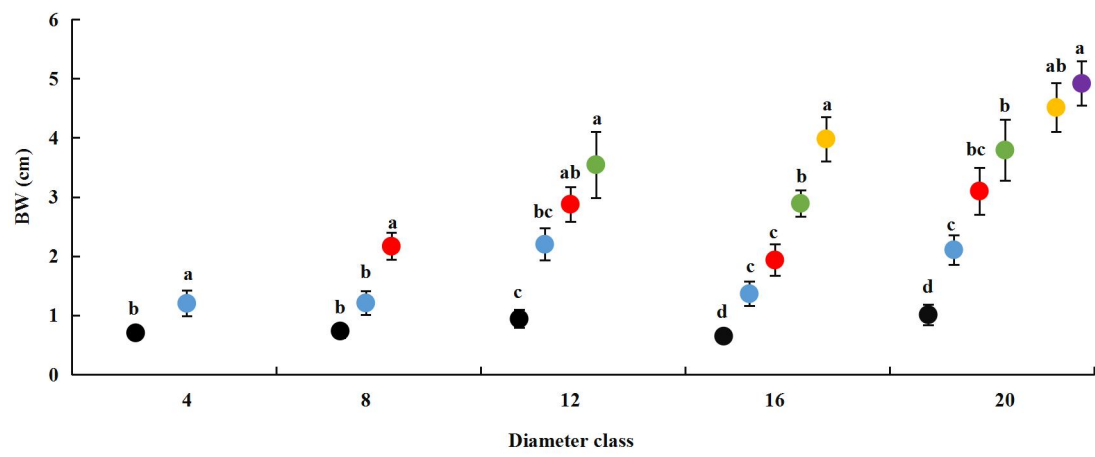

Supplement: Supplementary file 2 — Figure S2. Changes in leaf blade width across diameter class and sampling height scales in P. euphratica. Black dots indicate sampling height at 2 m, blue dots indicate 4 m, red dots indicate 6 m, green dots indicate 8 m, yellow dots indicate 10 m, purple dots indicate 12 m. [file PLB-22-366-s002.pdf]

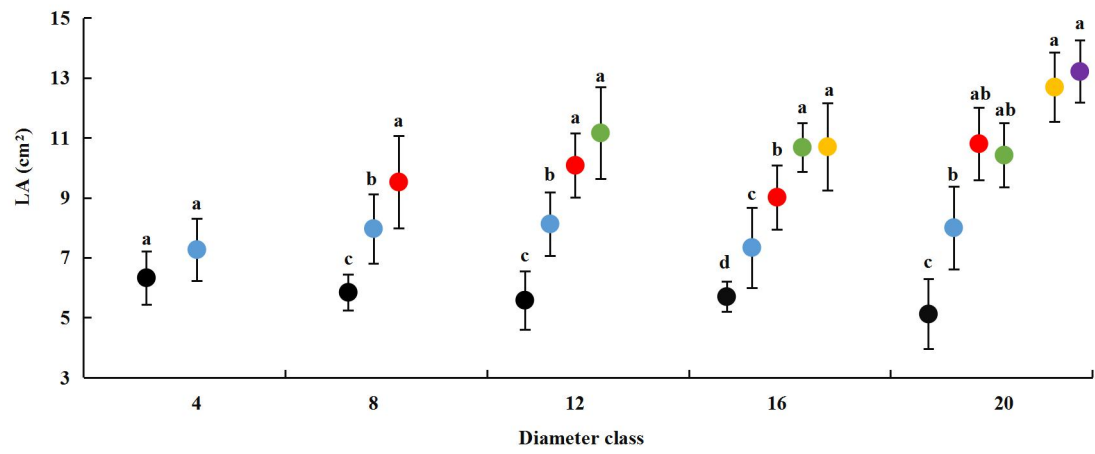

Supplement: Supplementary file 3 — Figure S3. Changes in leaf area across diameter class and sampling height scales in P. euphratica. Black dots indicate sampling height at 2 m, blue dots indicate 4 m, red dots indicate 6 m, green dots indicate 8 m, yellow dots indicate 10 m, purple dots indicate 12 m. [file PLB-22-366-s003.pdf]

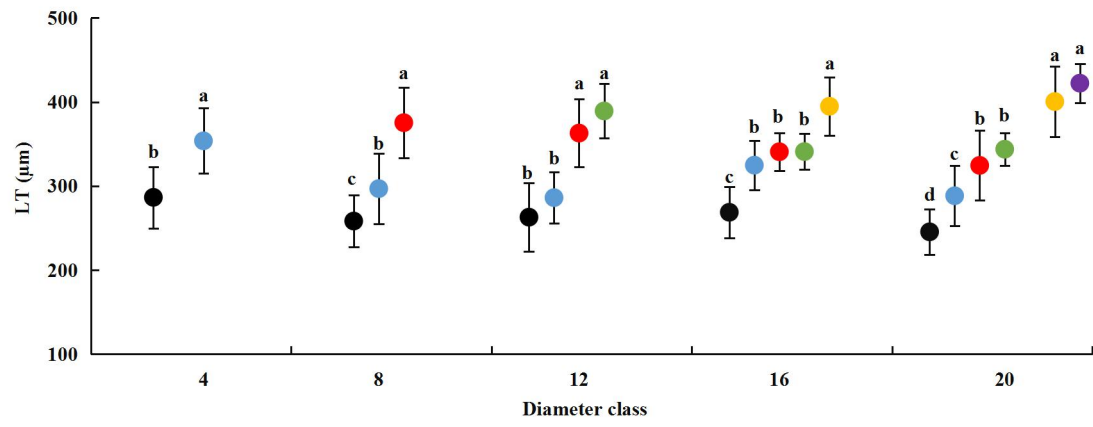

Supplement: Supplementary file 4 — Figure S4. Changes in leaf thickness across diameter class and sampling height scales in P. euphratica. Black dots indicate sampling height at 2 m, blue dots indicate 4 m, red dots indicate 6 m, green dots indicate 8 m, yellow dots indicate 10 m, purple dots indicate 12 m. [file PLB-22-366-s004.pdf]

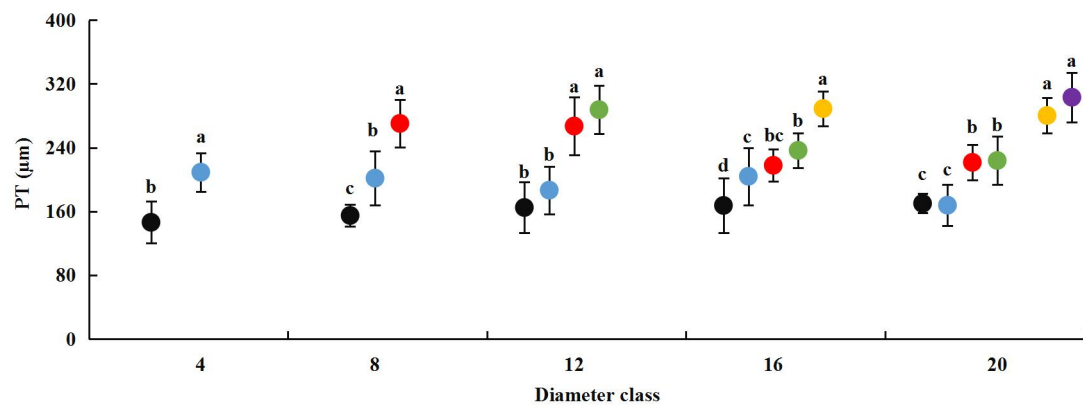

Supplement: Supplementary file 5 — Figure S5. Changes in leaf palisade thickness across diameter class and sampling height scales in P. euphratica. Black dots indicate sampling height at 2 m, blue dots indicate 4 m, red dots indicate 6 m, green dots indicate 8 m, yellow dots indicate 10 m, purple dots indicate 12 m. [file PLB-22-366-s005.pdf]

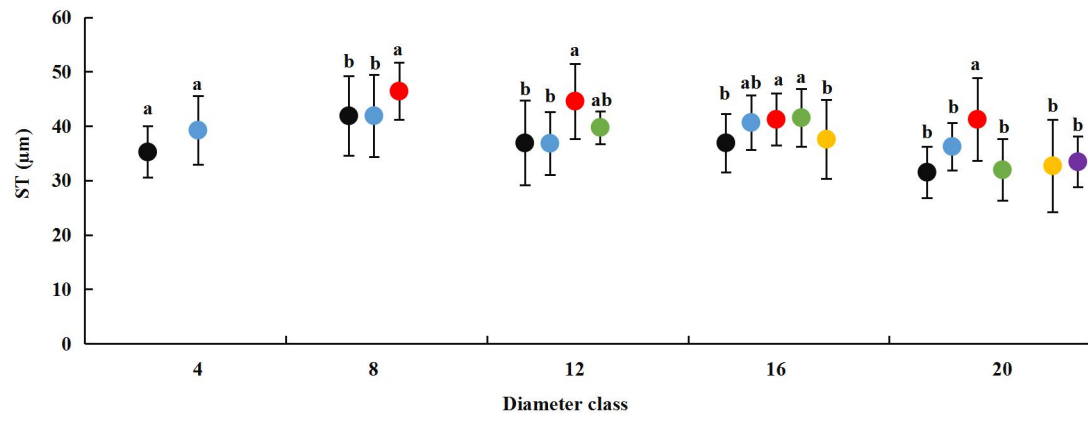

Supplement: Supplementary file 6 — Figure S6. Changes in leaf spongy tissue thickness across diameter class and sampling height scales in P. euphratica. Black dots indicate sampling height at 2 m, blue dots indicate 4 m, red dots indicate 6 m, green dots indicate 8 m, yellow dots indicate 10 m, purple dots indicate 12 m. [file PLB-22-366-s006.pdf]

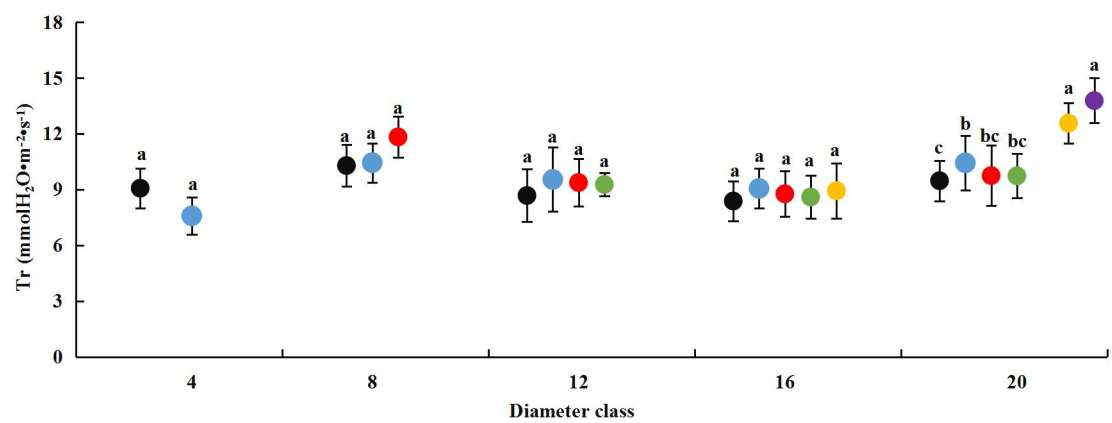

Supplement: Supplementary file 7 — Figure S7. Changes in leaf transpiration rate (E) across diameter class and sampling height scales in P. euphratica. Black dots indicate sampling height at 2 m, blue dots indicate 4 m, red dots indicate 6 m, green dots indicate 8 m, yellow dots indicate 10 m, purple dots indicate 12 m. [file PLB-22-366-s007.pdf]

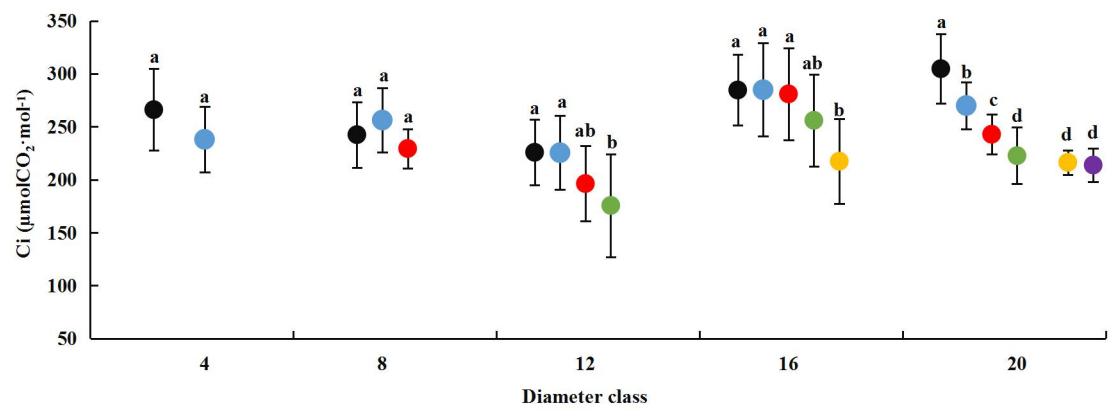

Supplement: Supplementary file 8 — Figure S8. Changes in leaf intercellular CO2 concentration (Ci) across diameter class and sampling height scales in P. euphratica. Black dots indicate sampling height at 2 m, blue dots indicate 4 m, red dots indicate 6 m, green dots indicate 8 m, yellow dots indicate 10 m, purple dots indicate 12 m. [file PLB-22-366-s008.pdf]

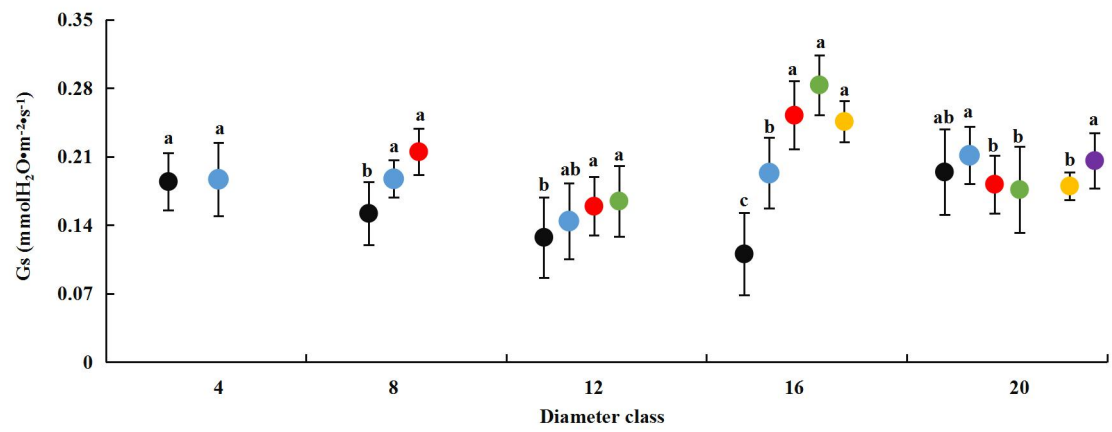

Supplement: Supplementary file 9 — Figure S9. Changes in leaf stomatal conductance (gs) across diameter class and sampling height scales in P. euphratica. Black dots indicate sampling height at 2 m, blue dots indicate 4 m, red dots indicate 6 m, green dots indicate 8 m, yellow dots indicate 10 m, purple dots indicate 12 m. [file PLB-22-366-s009.pdf]

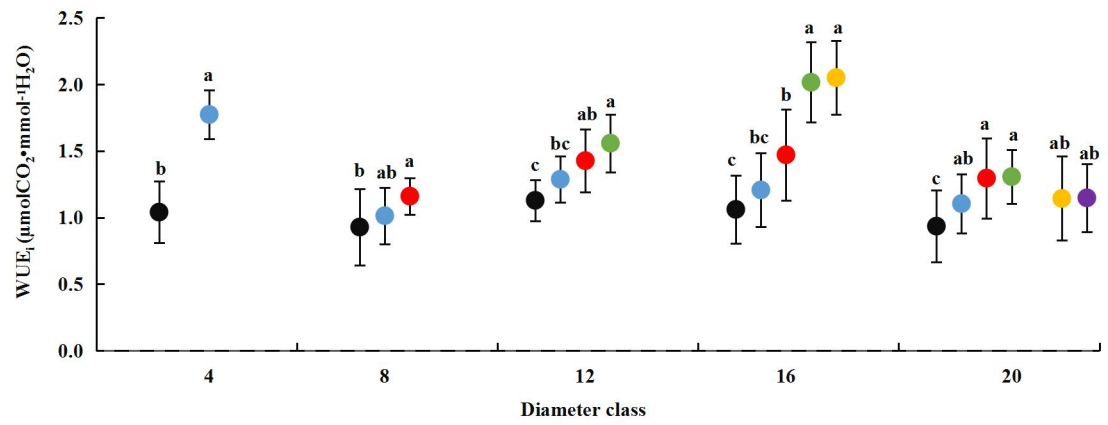

Supplement: Supplementary file 10 — Figure S10. Changes in leaf instantaneous water use efficiency (WUEi) across diameter class and sampling height scales in P. euphratica. Black dots indicate sampling height at 2 m, blue dots indicate 4 m, red dots indicate 6 m, green dots indicate 8 m, yellow dots indicate 10 m, purple dots indicate 12 m. [file PLB-22-366-s010.pdf]

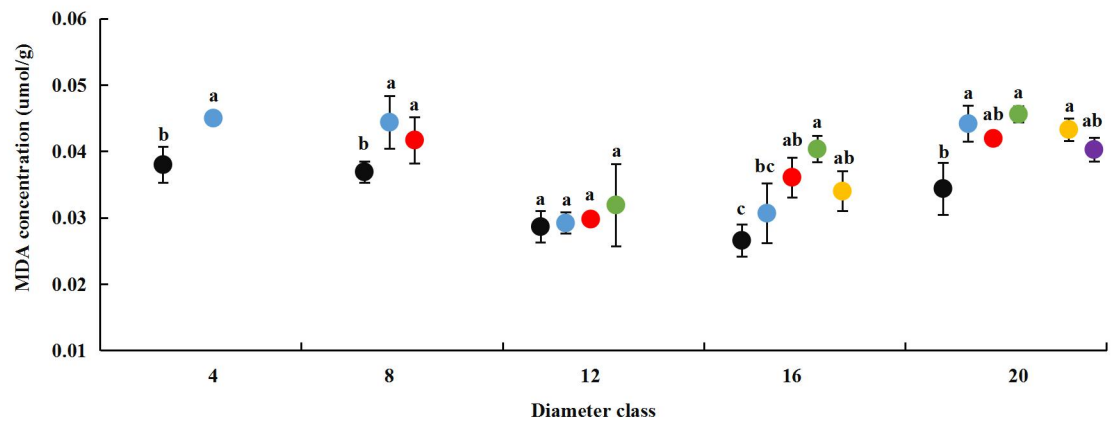

Supplement: Supplementary file 11 — Figure S11. Changes in leaf malondialdehyde (MDA) concentration across diameter class and sampling height scales in P. euphratica. Black dots indicate sampling height at 2 m, blue dots indicate 4 m, red dots indicate 6 m, green dots indicate 8 m, yellow dots indicate 10 m, purple dots indicate 12 m. [file PLB-22-366-s011.pdf]
