# Supplementary material for: Morphological, structural and physiological differences in heteromorphic leaves of Euphrates poplar during development stages and at crown scales
Source: Plant Biol (Stuttg). 2020 Jan 5;22(3):366–75. doi: 10.1111/plb.13078 (PMC7318281; doi:10.1111/plb.13078)
Supplement: Supplementary file 12 — Table S1. Information about the sampled trees. [file PLB-22-366-s012.pdf]

Table S1 Information about the sampled tree

Diameter class	ID			Diameter at breast height (cm)			Height of first branch（cm）			Tree height (m)		
	1	2	3	1	2	3	1	2	3	1	2	3
4	3001	1021	1034	5.21	5.83	5.33	148.43	158.62	149.32	5.03	5.02	5.37
8	2043	2092	1136	7.41	8.13	8.38	148.45	143.22	170.12	7.42	7.65	7.45
12	1028	2022	1041	12.78	13.32	13.56	141.37	147.30	117.74	9.50	8.90	8.77
16	1004	2098	1090	15.50	17.52	17.52	133.61	221.43	221.43	10.29	10.21	9.98
20	1070	1103	1102	20.40	21.50	21.50	193.32	214.75	214.75	12.10	13.17	13.49
