# Supplementary material for: Morphological, structural and physiological differences in heteromorphic leaves of Euphrates poplar during development stages and at crown scales
Source: Plant Biol (Stuttg). 2020 Jan 5;22(3):366–75. doi: 10.1111/plb.13078 (PMC7318281; doi:10.1111/plb.13078)
Supplement: Supplementary file 13 — Table S2. Correlation analysis among parameters related to morphological structure and physiology of heteromorphic leaves in P. euphratica. [file PLB-22-366-s013.pdf]

Table S2 Correlation analysis among parameters related to the morphological structure and physiology of heteromorphic leaves in *P. euphratica*

R	BL	BW	LI	LA	LT	LMA	PT	ST	CT	PSR	Pn	Tr	Gs	Ci	WUE_i	$\delta^{13}C$	Pro	MDA
BW	-0.45^{*}	1																
LI	0.57^{**}	-0.88^{**}	1															
LA	-0.35	0.93^{**}	-0.87^{**}	1														
LT	-0.53^{*}	0.73^{**}	-0.77^{**}	0.76^{*}	1													
LMA	-0.49^{*}	0.70^{**}	-0.61^{**}	0.59^{*}	0.67^{**}	1												
PT	-0.36	0.81^{**}	-0.74^{**}	0.79^{*}	0.92^{**}	0.71^{*}	1											
ST	-0.08	-0.26	0.03	-0.1	0.2	-0.3	0.01	1										
CT	-0.52^{*}	0.86^{**}	-0.78^{**}	0.78^{*}	0.61^{**}	0.79^{*}	0.66^{**}	-0.28	1									
PSR	-0.34	0.84^{**}	-0.73^{**}	0.81^{*}	0.70^{**}	0.74^{*}	0.81^{**}	-0.39	0.70^{**}	1								
Pn	-0.34	0.79^{**}	-0.75^{**}	0.72^{*}	0.84^{**}	0.74^{*}	0.88^{**}	0.02	0.65^{**}	0.69^{**}	1							
Tr	-0.29	0.47^{*}	-0.31	0.49^{*}	0.31	0.21	0.31	-0.34	0.19	0.47^{*}	0.15	1						
Gs	-0.54^{*}	0.38	-0.54^{*}	0.23	0.46^{*}	0.42	0.35	0.08	0.32	0.2	0.57^{**}	0.07	1					
Ci	-0.13	-0.64^{**}	0.48^{*}	-0.77	-0.60^{**}	-0.42	-0.70^{**}	-0.01	-0.49^{*}	-0.67^{*}	-0.61^{*}	-0.28	0.17	1				
WUE_i	-0.24	0.48^{*}	-0.54^{*}	0.38	0.58^{**}	0.57^{*}	0.58^{**}	0.23	0.53^{*}	0.32	0.82^{**}	-0.42	0.56^{**}	-0.33	1			
$\delta^{13}C$	-0.42	0.64^{**}	-0.56^{**}	0.49^{*}	0.76^{**}	0.76^{*}	0.82^{**}	-0.13	0.49^{*}	0.69^{**}	0.82^{**}	0.28	0.59^{**}	-0.33	0.58^{**}	1		
Pro	-0.54^{*}	0.52^{*}	-0.62^{**}	0.53^{*}	0.69^{**}	0.79^{*}	0.60^{**}	-0.02	0.66^{**}	0.53^{*}	0.63^{**}	0.01	0.41	-0.29	0.58^{**}	0.58^{*}	1	
MDA	-0.4	0.22	-0.35	0.25	0.29	0.04	0.08	0.23	-0.01	0.17	0.16	0.38	0.43	-0.02	0.03	0.16	0.05	1

Note: N=220, * $p<0.05$, ** $p<0.01$; BL: Blade length; BW: Blade width; LI:Leaf index; LA: Leaf area; LT: Leaf thickness; LMA: Leaf mass per area; PT: Palisade tissue thickness; ST: Sponge tissue thickness; CT: Cuticle thickness; PSR: Palisade tissue/sponge tissue ratio; Pn: Photosynthetic rate; Tr: Transpiration rate; Gs: Stomatal conductance; Ci: Intercellular CO_2 concentration; WUEi:Instantaneous water use efficiency; Pro:Proline; MDA: Malondialdehyde.
