# Supplementary material for: Morphological, structural and physiological differences in heteromorphic leaves of Euphrates poplar during development stages and at crown scales
Source: Plant Biol (Stuttg). 2020 Jan 5;22(3):366–75. doi: 10.1111/plb.13078 (PMC7318281; doi:10.1111/plb.13078)
Supplement: Supplementary file 14 — Table S3. Comparisons of morphology in heteromorphic leaves in sampling height gradients in the same diameter class and across diameter class at the same height. [file PLB-22-366-s014.pdf]

Table S3 The comparisons of morphology on heteromorphic leaves in sampling height gradients at the same diameter class and across diameter class at the same height

Diameter class	Sampling height（m）	BL（cm）	BW（cm）	LI	LA（cm^2）	LT（μm）	LMA
4	2	5.00 ± 0.57 a D	0.70 ± 0.18 b B	8.10 ± 1.40 a A	6.33 ± 0.89 a A	286.6 ± 36.56 b A	0.092 ± 0.012 a B
	4	5.95 ± 0.35 a ABC	1.20 ± 0.47 a B	4.70 ± 1.70 b B	7.26 ± 1.02 a C	353.69 ± 38.89 a A	0.099 ± 0.016 a A
8	2	7.29 ± 0.43 a A	0.73 ± 0.11 b B	7.90 ± 1.53 a A	5.84 ± 0.59 a AB	258.29 ± 30.98 c A	0.087 ± 0.009 a B
	4	6.31 ± 0.63 b AB	1.20 ± 0.19 b B	5.51 ± 0.64 b A	7.96 ± 1.15 b B	296.86 ± 41.56 b B	0.096 ± 0.022 a A
	6	5.94 ± 0.71 b B	2.16 ± 0.52 a B	3.09 ± 0.68 c AB	9.52 ± 1.54 a B	375.28 ± 41.98 a A	0.092 ± 0.025 a B
12	2	7.05 ± 0.55 a AB	0.94 ± 0.19 c A	7.23 ± 1.31 a B	5.58 ± 0.96 c B	262.91 ± 40.54 b A	0.075 ± 0.016 c C
	4	6.68 ± 0.66 ab A	2.20 ± 0.77 bc A	3.53 ± 1.16 b C	8.12 ± 1.06 b A	296.11 ± 30.39 b B	0.105 ± 0.005 b A
	6	6.53 ± 0.32 b A	2.28 ± 0.69 ab A	2.55 ± 0.71 b B	10.07 ± 1.07 a B	362.90 ± 40.52 a A	0.096 ± 0.012 b B
	8	6.46 ± 0.24 b A	3.54 ± 0.55 a A	2.42 ± 0.49 b A	11.16 ± 1.53 a A	389.19 ± 32.44 a A	0.147±0.021 a A
16	2	6.70 ± 0.60 a C	0.64 ± 0.08 d B	7.11 ± 1.22 a B	5.70 ± 0.49 d B	268.73 ± 30.37 c A	0.105 ± 0.015 c A
	4	6.46 ± 0.91 a C	1.36 ± 0.30 c B	4.70 ± 1.51 b B	7.33 ± 1.33 c B	324.74 ± 29.34 b A	0.105 ± 0.012 c A
	6	5.29 ± 0.48 b C	1.93 ± 0.26 c B	3.15 ± 0.39 c A	9.01 ± 1.07 b C	340.74 ± 22.45 b A	0.122 ± 0.017 b A
	8	5.25 ± 0.41 b B	2.89 ± 0.81 b A	2.17 ± 0.26 d A	10.67 ± 0.81 a A	340.91 ± 21.28 b B	0.129 ± 0.016 ab B
	10	5.39 ± 0.36 b A	3.98 ± 0.70 a A	1.73 ± 0.44 d A	10.69 ± 1.45 a A	394.79 ± 34.65 a A	0.143 ± 0.016 a A
20	2	6.65 ± 0.42 a B	1.01 ± 0.16 d AB	7.04 ± 1.22 a B	5.12 ± 1.16 c B	245.48 ± 26.75 d B	0.087 ± 0.003 c B
	4	5.53 ± 0.68 b BC	2.10 ± 0.55 c A	3.29 ± 1.41 b C	7.99 ± 1.38 bc C	288.37 ± 35.77 c B	0.093 ± 0.011 c A
	6	5.26 ± 0.80 b C	3.09 ± 0.39 b A	1.86 ± 0.51 cd C	10.80 ± 1.20 ab A	324.48 ± 41.34 b A	0.093 ± 0.009 c B
	8	5.57 ± 0.73 b B	3.79 ± 0.51 b A	2.27 ± 0.54 c A	10.42 ± 1.07 a A	343.72 ± 19.34 b B	0.116 ± 0.005 b B
	10	5.27 ± 0.58 b A	4.51 ± 0.90 ab A	1.85 ± 0.70 cd A	12.68 ± 1.15 a A	400.40 ± 42.23 a A	0.134 ± 0.009 a A
	12	4.39 ± 0.68 c	4.91 ± 0.82 a	1.41 ± 0.57 d	13.20 ± 1.04 a	422.25 ± 23.24 a	0.136 ± 0.011 a

Note: Lowercase letter(s) indicate significant differences among the different sampling heights of the same class. uppercase letters indicate significant differences among the same height of different class, *P*<0.05; BL: Blade length; BW: Blade width; LI:Leaf index; LA: Leaf area; LT: Leaf thickness; LMA: Leaf mass per area.
