# Supplementary material for: Morphological, structural and physiological differences in heteromorphic leaves of Euphrates poplar during development stages and at crown scales
Source: Plant Biol (Stuttg). 2020 Jan 5;22(3):366–75. doi: 10.1111/plb.13078 (PMC7318281; doi:10.1111/plb.13078)
Supplement: Supplementary file 15 — Table S4. Comparisons of anatomical structure of heteromorphic leaves in sampling height gradients in the same diameter class and across diameter class at the same height. [file PLB-22-366-s015.pdf]

Table S4 The comparisons on anatomic structure of heteromorphic leaves in sampling height gradients at the same diameter class and across diameter class at the same height

Diameter class	Sampling height（m）	PT（μm）	ST（μm）	PSR	CT（μm）
4	2	146.32 ± 26.13 b A	35.25 ± 4.72 a B	1.97 ± 0.34 a B	4.59 ± 0.65 a C
	4	209.14 ± 24.31 a A	39.25 ± 6.26 a A	1.96 ± 0.41 a C	4.62 ± 0.88 a B
8	2	154.89 ± 14.06 c A	41.90 ± 7.35 b A	1.89 ± 0.32 b B	4.62 ± 0.56 b C
	4	201.69 ± 34.41 b A	41.91 ± 7.59 b A	2.89 ± 0.55 a A	4.66 ± 0.55 ab B
	6	270.33 ± 30.10 a A	46.43 ± 5.28 a A	3.16 ± 0.46 a A	4.85 ± 0.69 a B
12	2	164.86 ± 32.51 b A	36.91 ± 7.77 b B	2.34 ± 0.42 c A	6.12 ± 0.89 c A
	4	186.62 ± 30.21 b AB	36.84 ± 5.80 b B	2.52 ± 0.38 c B	6.69 ± 0.48 b A
	6	267.03 ± 36.62 a A	44.59 ± 6.87 a AB	3.15 ± 0.65 b A	6.79 ± 0.56 b A
	8	287.58 ± 30.17 a A	39.77 ± 3.01 ab A	3.50 ± 0.57 a AB	7.98 ± 0.86 a A
16	2	167.44 ± 34.25 d A	36.93 ± 5.36 b B	2.42 ± 0.47 c B	5.41 ± 0.56 c B
	4	204.05 ± 36.34 c A	40.67 ± 5.00 ab A	2.47 ± 0.54 c B	6.39 ± 1.03 b A
	6	217.78 ± 20.14 bc B	41.24 ± 4.75 a B	2.67 ± 0.38 c B	7.36 ± 1.00 a A
	8	236.55 ± 22.37 b B	41.55 ± 5.34 a A	3.12 ± 0.51 b B	7.36 ± 0.78 a B
	10	288.92 ± 21.74 a A	37.59 ± 7.22 b A	3.44 ± 0.46 a A	7.53 ± 1.03 a A
20	2	170.00 ± 12.31 c A	31.52 ± 4.75 b C	2.08 ± 0.33 c B	5.17 ± 0.70 d B
	4	167.75 ± 26.03 c B	36.26 ± 4.40 b B	2.41 ± 0.50 bc B	6.30 ± 0.66 c A
	6	221.45 ± 22.44 b B	41.25 ± 7.62 a B	2.79 ± 0.49 b B	7.21 ± 1.02 b A
	8	223.82 ± 30.05 b B	31.97 ± 5.67 b B	3.79 ± 0.37 a A	7.15 ± 1.17 b B
	10	280.24 ± 22.13 a A	32.71 ± 8.50 b B	3.69 ± 0.52 a A	7.90 ± 1.13 a A
	12	303.13 ± 31.75 a	33.45 ± 4.63 b	3.65 ± 0.39 a	8.28 ± 0.96 a

Note: Lowercase letter(s) indicate significant differences among the different sampling heights of the same class. uppercase letters indicate significant differences among the same height of different class, $P<0.05$; PT: Palisade tissue thickness; ST: Sponge tissue thickness; CT: Cuticle thickness; PSR: Palisade tissue/sponge tissue ratio.
