# Supplementary material for: Morphological, structural and physiological differences in heteromorphic leaves of Euphrates poplar during development stages and at crown scales
Source: Plant Biol (Stuttg). 2020 Jan 5;22(3):366–75. doi: 10.1111/plb.13078 (PMC7318281; doi:10.1111/plb.13078)
Supplement: Supplementary file 16 — Table S5. Comparisons of gas exchange capacity of heteromorphic leaves in sampling height gradients in the same diameter class and across diameter class at the same height. [file PLB-22-366-s016.pdf]

Table S5 The comparisons on gas exchange capacity of heteromorphic leaves in sampling height gradients at the same diameter class and across diameter class at the same height

Diameter class	Sampling height（m）	Pn（$\mu molCO_2 \cdot m^{-2} \cdot s^{-1}$）	Tr（$mmolH_2O \bullet m^{-2} \bullet s^{-1}$）	Ci（$\mu mol\ CO_2 \cdot mol^{-1}$）	Gs（$mmolH_2O \bullet m^{-2} \bullet s^{-1}$）
4	2	8.51 ± 1.65 b B	9.06 ± 1.05 a B	266.05 ± 38.53 a ABC	0.184 ± 0.029 a A
	4	12.90 ± 1.77 a A	7.58 ± 1.04 a B	238.00 ± 31.02 a CD	0.186 ± 0.037 a A
8	2	8.78 ± 2.05 b B	10.28 ± 1.11 a A	242.43 ± 30.93 a BC	0.152 ± 0.032 b AB
	4	10.37 ± 2.10 b B	10.44 ± 1.05 a A	256.22 ± 30.24 a BC	0.187 ± 0.019 a A
	6	13.82 ± 2.44 a A	11.82 ± 1.09 a A	229.36 ± 18.45 a B	0.215 ± 0.023 a B
12	2	9.69 ± 1.51 c A	8.67 ± 1.41 a B	225.79 ± 31.16 a C	0.127 ± 0.041 b B
	4	11.60 ± 1.80 b AB	9.53 ± 1.72 a A	225.40 ± 34.76 a D	0.144 ± 0.038 ab B
	6	13.15 ± 2.33 a A	9.37 ± 1.26 a B	196.26 ± 35.44 ab C	0.159 ± 0.029 a C
	8	14.38 ± 1.88 a B	9.26 ± 0.61 a A	175.60 ± 48.39 b C	0.164 ± 0.036 a B
16	2	8.52 ± 2.17 c B	8.37 ± 1.06 a B	284.59 ± 33.30 a AB	0.110 ± 0.041 c B
	4	10.15 ± 1.42 bc B	9.05 ± 1.07 a A	284.88 ± 44.11 a A	0.193 ± 0.036 b A
	6	11.63 ± 2.34 b B	8.76 ± 1.23 a B	280.93 ± 43.35 a A	0.252 ± 0.034 a A
	8	16.34 ± 1.15 a A	8.59 ± 1.16 a A	255.96 ± 43.61 a B	0.283 ± 0.030 a A
	10	17.58 ± 2.02 a A	8.92 ± 1.48 a B	217.36 ± 39.96 b A	0.245 ± 0.021 a A
20	2	7.97 ± 2.88 e B	9.46 ± 1.08 c B	304.73 ± 32.61 a A	0.194 ± 0.043 ab A
	4	9.95 ± 1.63 d B	10.42 ± 1.46 bc A	269.94 ± 22.20 b AB	0.211 ± 0.029 a A
	6	11.02 ± 1.15 cd B	9.74 ± 1.61 b B	242.83 ± 18.66 c B	0.181 ± 0.027 b C
	8	11.83 ± 1.29 bc C	9.73 ± 1.19 b A	222.61 ± 26.65 d A	0.176 ± 0.043 b B
	10	12.83 ± 0.93 b B	12.56 ± 1.09 ab A	216.27 ± 11.61 d A	0.180 ± 0.014 b B
	12	14.49 ± 1.38 a	13.77 ± 1.20 a	213.76 ± 15.89 d	0.205 ± 0.028 a

Note: Lowercase letter(s) indicate significant differences among the different sampling heights of the same class. uppercase letters indicate significant differences among the same height of different class, P<0.05. Pn: Photosynthetic rate; Tr: Transpiration rate; Gs: Stomatal conductance; Ci: Intercellular CO_2 concentration.
