# Supplementary material for: Morphological, structural and physiological differences in heteromorphic leaves of Euphrates poplar during development stages and at crown scales
Source: Plant Biol (Stuttg). 2020 Jan 5;22(3):366–75. doi: 10.1111/plb.13078 (PMC7318281; doi:10.1111/plb.13078)
Supplement: Supplementary file 17 — Table S6. Comparisons of water use efficiency of heteromorphic leaves in sampling height gradients in the same diameter class and across diameter class at the same height. [file PLB-22-366-s017.pdf]

Table S6 The comparisons on water use efficiency of heteromorphic leaves in sampling height gradients at the same diameter class and across diameter class at the same height

Diameter class	Sampling height（m）	WUEi（μmolCO_2•$mmol^{-1}H_2O$）	б^{13}C
4	2	1.04 ± 0.23 b A	-29.38 ± 0.63 a C
	4	1.77 ± 0.18 a A	-28.27 ± 0.61 a A
8	2	0.93 ± 0.20 b B	-28.38 ± 0.57 a A
	4	1.01 ± 0.21 ab B	-28.22 ± 0.46 a A
	6	1.16 ± 0.13 a B	-27.14 ± 0.58 a A
12	2	1.13 ± 0.15 c A	-29.37 ± 0.46 b C
	4	1.29 ± 0.17 bc B	-28.82 ± 0.32 ab B
	6	1.43 ± 0.23 ab A	-28.01 ± 0.33 ab B
	8	1.56 ± 0.21 a B	-27.55 ± 0.57 a AB
16	2	1.06 ± 0.25 c A	-28.21 ± 0.26 c A
	4	1.27 ± 0.27 bc B	-27.80 ± 0.57 bc A
	6	1.47 ± 0.34 b A	-27.60 ± 0.65 a AB
	8	2.02 ± 0.30 a A	-27.13 ± 0.31 ab A
	10	2.05 ± 0.27 a A	-26.65 ± 0.15 a A
20	2	0.93 ± 0.17 c B	-28.86 ± 0.50 bc B
	4	1.10 ± 0.22 ab B	-29.08 ± 0.42 c B
	6	1.30 ± 0.30 a AB	-28.44 ± 0.50 abc B
	8	1.31 ± 0.20 a B	-27.97 ± 0.42 abc B
	10	1.14 ± 0.31 ab B	-27.31 ± 0.51 ab B
	12	1.15 ± 0.25 ab	-26.90 ± 0.66 a

Note: Lowercase letter(s) indicate significant differences among the different sampling heights of the same class. uppercase letters indicate significant differences among the same height of different class, *P*<0.05; WUEi:Instantaneous water use efficiency.
