# Supplementary material for: Morphological, structural and physiological differences in heteromorphic leaves of Euphrates poplar during development stages and at crown scales
Source: Plant Biol (Stuttg). 2020 Jan 5;22(3):366–75. doi: 10.1111/plb.13078 (PMC7318281; doi:10.1111/plb.13078)
Supplement: Supplementary file 18 — Table S7. Comparisons of physiological characteristics of heteromorphic leaves in sampling height gradients in the same diameter class and across diameter class at the same height. [file PLB-22-366-s018.pdf]

Table S7 The comparisons on physiological characteristics of heteromorphic leaves in sampling height gradients at the same diameter class and across diameter class at the same height

Diameter class	Sampling height（m）	Pro（ug/g）	MDA（umol/g）
4	2	11.47 ± 2.71 a A	0.037 ± 0.007 b A
	4	12.39 ± 2.32 a A	0.045 ± 0.002 a A
8	2	6.51 ± 1.15 b B	0.036 ± 0.004 b A
	4	8.34 ± 1.16 b C	0.044 ± 0.011 a A
	6	10.61 ± 2.22 a A	0.041 ± 0.010 a A
12	2	7.94 ± 1.32 c B	0.028 ± 0.007 a B
	4	10.04 ± 1.22 b B	0.029 ± 0.004 a B
	6	11.23 ± 1.66 b A	0.029 ± 0.003 a C
	8	14.67 ± 1.80 a A	0.031 ± 0.008 a C
16	2	10.80 ± 0.80 b A	0.026 ± 0.007 c B
	4	11.73 ± 1.25 ab A	0.030 ± 0.012 bc B
	6	11.70 ± 1.23 ab A	0.036 ± 0.008 ab B
	8	13.76 ± 1.85 a A	0.040 ± 0.006 a B
	10	12.04 ± 2.24 ab A	0.033 ± 0.009 bc B
20	2	10.26 ± 1.56 ab A	0.034 ± 0.011 b A
	4	9.47 ± 1.44 b B	0.044 ± 0.007 a A
	6	9.00 ± 1.97 b B	0.041 ± 0.003 bc A
	8	11.63 ± 1.75 ab B	0.045 ± 0.003 a A
	10	13.39 ± 1.83 a A	0.043 ± 0.005 a A
	12	12.60 ± 1.73 ab	0.040 ± 0.005 ab

Note: Lowercase letter(s) indicate significant differences among the different sampling heights of the same class. uppercase letters indicate significant differences among the same height of different class, *P*<0.05; Pro:Proline; MDA: Malondialdehyde.
